# Original Communications

**Published:** 1855-07

**Authors:** WM. R. Abbey

**Affiliations:** Of the firm of Chas. Abbey & Sons. Philadelphia


### ARTICLE VII.

*To the Editors of American Journal of Dental Science.*

Gentlemen:

I find in your number for April last, an article, (vi,) entitled, "Report on Dental Progress," in which the author, A. M. Leslie, D. D. S., presents views and makes deductions in relation to "Abbey's foil," which are evidently based upon erroneous impressions; with a desire to correct which, I would ask permission to occupy a short space in your Journal.

Dr. Dwinelle appears, (at some previous time,) to have instituted certain comparisons between different makes of foil and sponge gold, and needing a familiar standard, had adopted Abbey's foil. Dr. Leslie objects to the choice, as follows: "Undoubtedly Dr. Dwinelle is right when he says, 'pure gold is of a uniform color throughout the world,' but he falls into an error in which he will find abundance of company when he gives Abbey's foil *as a standard of color* to test pure gold by throughout the world. This can be shown in various ways; 1st, Abbey's foil used to be, and we suppose yet is, occasionally *variable in color;* 2d, that peculiar red color it possesses is only superficial; melt a leaf or two of it with the blow-pipe, and you will find it range in color with some of 'the paler foils of the market.' It wears a livery which has been taken by many besides the doctor to indicate its royalty.* * * What this coloring matter is, may be difficult to determine, but of one thing be assured, *it is only present when iron is made use of in the process of purification*, and need not be then if the gold is *perfectly washed.*"

An *examination* of our foil will convince any one at once that it is *not* of a "peculiar *red* color." That in color it is deeper than most other foils, I admit, but this arises from no artificial or "superficial" process, or neglect through ignorance or inattention in any part of the primary "process of purification," but is a legitimate result of strictly scientific manipulations, carefully and properly performed, and is offered and claimed to be a natural genuine fine gold color. The writer has, for the last five years, personally performed all that appertains to the refining and melting department in the business of our firm, and can confidently vouch for the fact that the particular process of "washing off," is conducted with a faithful and patient use of such chemical and other means as are known to be best applicable to the purpose of removing introduced or formed in the general process of refining, and for the above mentioned period at least, to his personal knowledge, Abbey's foil has not been "red."

Iron introduced into or permitted to remain, (as the Dr. suggests,) in fine gold, has the effect of coloring it an *unmistakable* red, either entire or in spots, according to the quantity of it present, and this discoloration is more or less visible in all stages of the preparation of the foil, as well in the bar as in the foil. Hence, if Abbey's foil *is* discolored or "red," (as per Dr. Leslie,) the "button" formed from "melting a leaf or two," will partake of the same shade of red. If it is *not* discolored "superficially" or otherwise, but is as I assert, natural and genuine in its color, the "button" will be slightly paler than the foil, perhaps almost imperceptibly so; this latter result I will proceed to explain, and in so doing, will at same time, cover the "objection" of Dr. Leslie, that "Abbey's foil is occasionally variable in color." Fine gold has the property, (one not generally known,) of becoming slightly deeper or darker in color as it is reduced in thickness, the difference in the shade being rendered more distinct by annealing. Thus the thinnest and the thickest foil, (say Nos. 4 and 10,) though made from the same bar and undergoing precisely same treatment in all respects, will differ in color slightly, the thinnest being the deepest or darkest shade. If similar Nos. of Abbey's foil are compared, (say No. 4 with No. 4, No. 5 with No. 5, &c.,) there will be found a uniform agreement in color, though a slight difference in shade may be discovered between the extreme Nos., (4 and 10,) for the reason above given. Were the complete foil and the original bar placed in contrast, the difference in shade would be quite obvious. I trust I have made clear, how our foil may be uniform in its color in one sense, though at same time "variable" in another sense, and also why the melted "button" is a shade paler than "the leaf or two of foil," of which it is composed.

Dr. Leslie alludes to the great adhesive quality that is exhibited in certain gold foil, even to the extent in some, "that simply the weight of one leaf laid upon another would forever unite them at the points of contact," and remarks further,

"It will never be found to possess the deep color of Abbey's foil, as when that is present in sufficient quantity, *it cannot be made to adhere.*"

The doctor, not only laboring under a misapprehension in attributing to Abbey's foil a "peculiar red color," and also in his explanation of the cause of whatever color it does possess, is again in error, in the idea thrown out here, that no adhesiveness can be found in Abbey's foil, *because* of the deep color. Every ounce of fine gold, (notwithstanding the "deep color,") passing through my hands, (in my capacity before mentioned,) has that adhesive quality of which the doctor speaks, to all the extent that its most ardent advocate would desire, although it is not to be found to an undue extent in our foil as sold. Our business correspondence embraces many of the most prominent dentists in the country, and their almost unanimous experience as centered upon us, together with our own observations, have tended to convince us that great adhesive foil is not adapted to the wants of the dental profession, or at least that portion who use our foil, and we have, consequently, acted upon this conviction, and been well sustained in it. The general tenor of their remarks have been, that in using the adhesive foil, (and there is no scarcity of it,) the various surfaces of the foil as prepared for filling, become firmly united either before or immediately upon the application of the instrument, so that the effort to pack the filling and break down the angles or corners, is one of great difficulty and generally results in the gold becoming hard and immovable, long before it has been made solid or been adapted to the irregularities or inequalities of the walls of the cavity, and is liable not only to admit the juices of the mouth between the filling and the walls, but is apt eventually to become loose and drop out.

Of course I do not presume to enter into a discussion so entirely within the province of the dental profession, and I only present this brief summary (in my own language,) of the views and experience of the mass of our correspondents, as expressed to us, for the purpose of showing why our foil does not exhibit great adhesiveness. It is probable that its uniform absence in our foil has impressed the doctor with the mistaken idea intimated in the passage last quoted.

WM. R. ABBEY,
Of the firm of Chas. Abbey & Sons.

*Philadelphia, May,* 1855.

